# Efficacy of Extractions of Iranian Native Plants against Main Malaria Vector, *Anopheles stephensi* in Iran for Making Appropriate Formulation for Disease Control

**Published:** 2019-12-31

**Authors:** Hassan Vatandoost, Fatemeh Nikpour, Ahmad Ali Hanafi-Bojd, Mohammadd Reza Abai, Mahnaz Khanavi, Abbas Hajiiakhondi, Ahmad Raesi, Jalil Nejati

**Affiliations:** 1 Department of Medical Entomology and Vector Control, School of Public Health, Tehran University of Medical Sciences, Tehran, Iran; 2 Department of Chemical Pollutants and Pesticides, Institute for Environmental Research, Tehran University of Medical Sciences, Tehran, Iran; 3 Department of Pharmacognosy, School of Pharmacy, Tehran University of Medical Sciences, Tehran, Iran

**Keywords:** Plants, Malaria vector, Pesticide, Iran

## Abstract

**Background:**

Malaria is the main vector–borne disease worldwide. There are several reports of insecticide resistant in malaria vectors worldwide due to using different insecticides. The aim of this study was to evaluate different native plant extortions against main malaria vector, *Anopheles stephensi* in Iran for choosing the appropriate plant for formulation and use for vector control.

**Methods:**

The larvae of *An. stephensi* were reared in insectary, extraction of plants were carried out at department of Pharmacology. The standard WHO method for biological tests was used for calculation of LC_50_ and LC_90_. Probit regration lines were plotted for calculation of LC_50_ and LC_90_.

**Results:**

In this study several plants including: *Mentha spicata*, *Cymbopogon olivieri*, *Azadirachta indica*, *Melia azedarach*, *Lagetes minuta*, *Calotropis procera*, *Eucalyptus camaldulensis*, *Cupressus arizonica*, *Thymus vulgaris*, *Lawsonia inermis*, *Cedrus deodara*, *Cionura erecta*, *Bunium persicum*, *Carum carvi*, *Artemisia dracunculus*, *Rosmarinus officinalis* were used. Results showed that *Mentha spicata* and *Eucalyptus camaldulensis*, had the lowest and highest LC_50_ respectively.

**Conclusion:**

Results indicated that *Mentha spicata* and *Eucalyptus camaldulensis,* had the lowest and highest LC_50_ respectively. Several other plant extract also showed significant mortality. The formulation of these plants should be prepared and evaluate at the field condition against malaria vectors.

## Introduction

Malaria is the most important mosquito-borne disease so that an estimated 212 million cases worldwide in 2015 out of them 3,800,000 cases estimated to be happen in Eastern Mediterranean Region (EMRO). It was estimated that 429,000 deaths from malaria occurred globally including 7300 cases in EMRO. The disease in the region had 291 million people at risk, and mostly reported from 5 countries: Sudan (36%), Pakistan (27%), Somalia (18%), Afghanistan (11%) and Yemen (8%) (1). There were an estimated 219 million cases and 435 000 related deaths in 2017 (2). Insecticide resistance is becoming a problem of global importance as it threatens the significant achievements in malaria control. Dramatic increase of insecticides use in malaria vector control projects has resulted to growing trend of insecticide resistance among mosquito vectors. Currently increased attention to pyrethroids as effective and low-risk insecticides has developed the risk of resistance to this group. Nowadays there are two main interventions in malaria control programs: indoor residual spraying (IRS) and long-lasting insecticidal nets (LLINs). For both methods, pyrethroids are used and this will increase the pressure of selection for resistance. As a result, there are reports indicating resistance to pyrethroids in malaria vectors of the EMRO region in recent years (3). Data are still limited and difficult to consolidate as many countries have not yet carried out adequate routine susceptibility tests, and malaria-free countries don’t usually do the tests.

During the last decade, three out of four insecticide classes were applied in malaria control activities in EMRO countries (4). Organophosphates including Malathion, Dichlorvos, Temephos and Fenitrothion with a total of 381 tones active ingredient had the highest use, followed by pyrethroids (Cypermethrin, Alpha-cypermethrin, Deltamethrin, Lambda-cyhalothrin, permethrin) and carbamates (Bendiocarb, Propoxur) with 157 and 30.6 tones active ingredient, respectively. In contrast of organo-phosphates, during these years using of pyrethriods and carbamates seems to be increased. Insecticide resistance is the selection of a heritable trait in an insect population that results in an insect-control product no longer performing as intended. Establishing the baseline of all plant evaluated against vector and conducting a comprehensive situation analysis is the starting point for overcome against resistance. This will require collecting available background data and, if necessary. Interpretation of the data must take into account. Countries should design a monitoring plan that includes data on vector distribution and relevant vector attributes for transmission and control, on susceptibility/ resistance to currently used insecticides, and on the quality of vector control interventions.

### Malaria in Iran

Malaria is one of the important infectious diseases in Iran with an average of about 15000 annual cases in the last decade. The most routes of malaria cases are immigration from Afghanistan and Pakistan to southern and southeastern areas of the country (Ministry of Health, annual reports). During the 2002–2017, 134,273 malaria cases were reported. The malaria incidence decreased from 0.24/1000 cases in 2002 to 0.01/1000 in 2017. From 2009 onward, the number of imported cases increased in comparison with the autochthonous and indigenous cases. Most cases were seen in males and people over 15 years of age. Moreover, the dominant registered reports were from rural areas. Most malaria cases were reported from the south and southeastern of Iran. *Plasmodium vivax* was the dominant species (5).

**Fig. 1.**
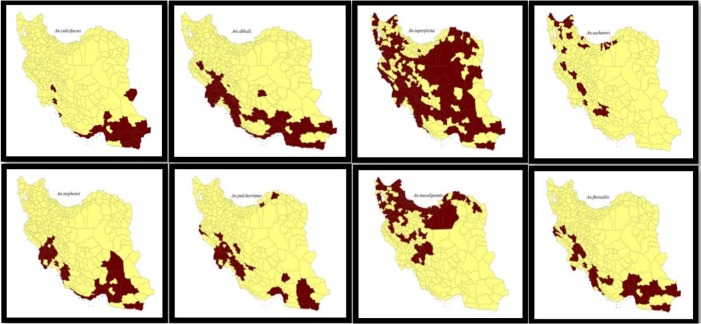
Map of Distribution of malaria vectors in Iran

There are several activities on different aspects of malaria in the country: including insecticide resistance monitoring (6–17), using bednets and long lasting impregnated nets (18–24). Recently resistance of *An. stephensi* to different insecticides in malarious areas of Iran has been reported (3). The last checklist of Iranian mosquitoes shows 31 Anopheles species including sibling, biological forms and genotypes, 17 out of them are reported to be included in malaria transmission. These vectors are considered as sibling, genotype and type forms. *Anopheles stephensi*, *An. culicifacies*, *An. fluviatilis*, *An. dthali* are the main vector species of south-eastern foci, while *An. sacharovi* and *An. maculipennis* are included in malaria transmission in northwest focus, and *An. superpictus* has wide distribution in all malaria foci of the country (Fig. 1).

Seasonal activity of Anopheline mosquitoes varies in different area due to environmental condition. It shows one peak in northwest especially in summer, however, there are two peaks of activity in coastal warm and humid region in the southern part of Iran with oriental epidemiological characteristics. The chemical control of vectors now is restricted to endemic malarious areas of south-eastern part of the country with Deltamethrin and residual spraying and long lasting permethrin impregnated nets (Olyset) for personal protection, while biological control is conducting by *Bacillus thuringiensis* as larvicide. Knowledge on insecticide resistance in target species is a basic requirement to guide insecticide use in malaria control programmes in local and global scales. The main criteria for susceptibility status, which are recommended by WHO, were considered. The results showed that there is resistance to DDT and dieldrin, indication of tolerance to some tested insecticides. Agriculture in Iran remains highly sensitive to climate developments; the country's most important crops are wheat, rice and other grains, sugar beet, fruits, nuts, cotton, and tobacco, which require the use of insecticides. So far different groups of insecticides are using for crops protection in the country. The main governmental use of insecticide in the health sector is their application for adult mosquito control. The campaign against malaria vectors started with organochlorines (DDT, dieldrin and BHC) during the 1960’s, followed by organophosphates (malathion and pirimiphos-methyl) for 2 decades from 1966 and continued with the carbamate, propoxur during 1977–1990, and then with pyrethroids including lambdacyhalothrin and Deltamethrin. Temephos, Reldan and pirimiphos-methyl was used for larviciding (Ministry of Heath of Iran)

## Materials and Methods

### Rearing of mosquito larvae

Rearing and maintaining mosquito larvae was carried out in the temperature of 29±2 ºC and relative humidity of 70±10% and Light dark cycle of 16h light and 8h was performed in Culicidae insectarium of the School of Public Health Tehran University of Medical Sciences. The larvae of the late 3rd stage or early 4th stage of *An. stephensi* were used for larvicidal tests. *Anopheles stephensi* larvae used in this study were obtained from the laboratory of the “School of Public Health and Institute of Health Research” Tehran University of Medical Sciences, Tehran, Iran. They were reared under insectary conditions at 25±1, 12/12h (light/dark) photo- period and 50–70% relative humidity and were fed with 10% sucrose solution. The late 3rd and early 4th instar larvae were used for the tests. The sucrose solution was withdrawn from the cage, 14h prior to the tests.

### Biological tests (larvicidal)

The standard WHO method for biological tests was used. The overall temperature of the lab (28 ºC), test period (24h) and the number of larvae (25 in each 400cc beaker) has to be constant. The best age range of the larvae for the tests are the larvae of the late 3^rd^ stage or early 4^th^ stage range and preferably dechlorinated water should be used in the tests. At least 5 logarithmic concentrations should be made of the EO. In order to find the suitable concentration first the concentrations should be chosen in a larger domain and based on the results the concentration domain becomes narrower. Usually the concentration in which has the 50% relative mortality and two concentrations lower than it and two concentrations upper than it are used to draw that regression line diagram. In each test 5 concentrations of pesticide and for each concentration 4 repetitions and in general 2 witnesses are considered.

### Statistical analysis

The test results after 24h were read as the following way: the number of alive larvae, the number of dead larvae, the number of moribund larvae, number of larvae and the total number and the results were used to draw the mortality tables. The mortal quantities of 50% and 90 % of EOs (LC_50_ and LC_90_) and the level of confidence of 95%, the equation of the regression line will be estimated using a regression probit analysis as described by Finney (25). When the mortality of the witness group is less than 5% then the resulted data of biometric tests have been correct but if the mortality of the witness group is between 5% to 20% they have to be corrected line. The percentage mortality was calculated using Abbot’s formula **(**26).

### Extraction

Solvent fractionation dried whole samples (300g) were extracted with 80% methanol (MeOH, 6×1.5l) in a percolator at room temperature for 2 weeks. The combined extract was concentrated to dryness under reduced pressure at 40 °C. The MeOH extract was successively dissolved in 100mL MeOH: H2O (7: 3) and extracted Mosquito rearing and evaluation with petroleum ether (4×200mL), chloroform (CHCl3, 4×200mL), H2O-saturated ethyl acetate (EtOAc, 4×200mL) and H2O-saturated n-butanol (n-BuOH, 4×200mL) in separatory funnel. Each fraction together with the remaining MeOH part after the solvent fractionation, were then evaporated to dryness under reduced pressure at 40 °C for the purpose of test fraction. All solvents were purchased from Merck (Merck, Darmstadt, Germany).

### List and Identification of plants

In this study the different extractions of the following Iranian native plants were evaluated against main malaria vector, *An. Stephensi*, *Mentha spicata*, *Cymbopogon olivieri*, *Azadirachta indica*, *Melia azedarach*, *Lagetes minuta*, *Calotropis procera*, *Eucalyptus camaldulensis*, *Cupressus arizonica*, *Thymus vulgaris*, *Lawsonia inermis*, *Cedrus deodara*, *Cionura erecta*, *Bunium persicum*, *Carum carvi*, *Artemisia dracunculus*, *Rosmarinus officinalis.*

## Results

**Fig. 2.**
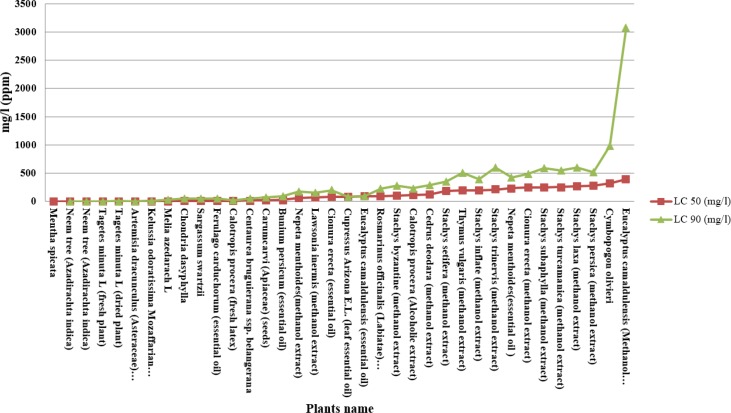
Efficacy of different plants extract against *Anopheles stephesni* at the LC_50_ and LC_90_ level

Results of efficacy of different Iranian native plants against malaria vector *An. stephensi* at the LC_50_ and LC_90_ levels are presented in Table 1 and Fig. 2. From these results it can be concluded that *Mentha spicata* and *Eucalyptus camaldulensis*, had the lowest and highest LC_50_ respectively.

**Table 1.** Efficacy of different plants extract against *Anopheles stephesni* at the LC_50_ and LC_90_ level

**Component name**	**LC_50_ (mg/l)**	**LC_90_ (mg/l)**	**Reference**
***Mentha spicata***	0.009		Hajiakjoondi A et al. (2000) (27)
***Cymbopogon olivieri***	321.90	983.6	Hadjiakhoondi A, et al. (2003) (28)
***Azadirachta indica***	0.35	1.81	Vatandoost H, et al. (2004) (29)
***Melia azedarach***	5.51	34.90	Hadjiakhoondi A, et al. (2006) (30)
***Tagetes minuta L* (dried plant)**	1.30	5.07	Hajiakhondi A, et al. (2008) (31)
***Tagetes minuta L* (fresh plant)**	1.05	3.83	Hajiakhondi A, et al. (2008) (31)
***Calotropis procera* (Alcoholic extract)**	109.71	234.61	Shahi M, et al. (2010) (32)
***Calotropis procera (* Fresh latex)**	13.06	23.53	Shahi M, et al. (2010) (32)
***Eucalyptus camaldulensis* (Methanol extract)**	397.75	3085.18	Sedaghat M, et al. (2010) (33)
***Eucalyptus camaldulensis* (essential oil)**	89.85	215.26	Sedaghat M, et al. (2010) (33)
**Cupressus Arizona E.L. (leaf essential oil)**	79.30	238.89	Sedaghat M, et al. (2011) (34)
***Centaurea bruguierana ssp.*** **belangerana**	15.70	48.34	Khanavi M, et al. (2011) (35)
***Sargassum swartzii***	11.75	53.47	Khanavi M, et al. (2011) (35)
***Chondria dasyphylla***	10.62	56.39	Khanavi M, et al. (2011) (35)
***Nepeta menthoides* (methanol extract)**	69.54	175.55	Khanavi M, et al. (2012) (36)
***Nepeta menthoides*** **(essential oil)**	234.35	419.86	Khanavi M, et al. (2012) (36)
***Kelussia odoratissima Mozaffarian* (essential oil)**	4.88	9.60	Vatandoost H, et al. (2012) (37)
***Thymus vulgaris* (methanol extract)**	191.33	503.98	Khanavi M et al. (2013) (38)
***Lawsonia inermis* (methanol extract)**	69.40	158.75	Khanavi M, et al. (2013) (38)
***Cedrus deodara* (methanol extract)**	128.04	292.87	Khanavi M, et al. (2013) (38)
***Stachys trinervis* (methanol extract)**	210.42	604.04	Khanavi M, et al. (2013) (38)
***Stachys inflate* (methanol extract)**	195.84	392.81	Khanavi M, et al. (2013) (38)
***Stachys setifera* (methanol extract)**	181.62	352.35	Khanavi M, et al. (2013) (38)
***Stachys laxa* (methanol extract)**	269.64	602.6	Khanavi M, et al. (2013) (38)
***Stachys persica* (methanol extract)**	282.80	515.94	Khanavi M, et al. (2013) (38)
***Stachys subaphylla* (methanol extract)**	252.60	592.37	Khanavi M, et al. (2013) (38)
***Stachys byzantine* (methanol extract)**	103.29	276.99	Khanavi M, et al. (2013) (38)
***Stachys turcamanica* (methanol extract)**	253.45	549.05	Khanavi M, et al. (2013) (38)
***Cionura erecta*** **(essential oil)**	77.30	199.58	Mozaffari E, et al. (2014) (39)
***Cionura erecta*** **(methanol extract)**	250.38	490.00	Mozaffari E, et al. (2014) (39)
***Ferulago carduchorum* (essential oil)**	12.78	47.43	Golfakhrabadi F, et al. (2015) (40)
***Bunium persicum* (essential oil)**	27.72	91.35	Sanei-Dehkordi A, et al (2016) (41)
***Carum carvi* (seeds)**	21.6	72.44	Torabi Pour H, et al. (2016) (42)
***Artemisia dracunculus* (branches and Leaves)**	1.33	4.12	Torabi Pour H, et al. (2016) (42)
***Rosmarinus officinalis* (branches and Leaves)**	93.22	229.29	Torabi Pour H, et al. (2016) (42)

## Discussion

Most botanical components are rapid acting and breakdown quickly in the environment. The extract of whole leaf and essential oil of some certain plants have been investigated against some public health pests. The use of botanical pesticide may help in reducing the environmental side effects by the synthetic insecticides. The results obtained suggest that the extracts of different Iranian native plants may be a promising as larvicide against *An. stephensi*. There are many researches in the field. In other investigation, Nathan et al. (2007) (43) reported that the larvicidal activity of essential oil from *Eucalyptus tereticornis* Sm. with LC_50_ and LC_90_ values were 23.8 and 63.9ppm respectively against *An. stephensi* larvae. There are some reports about the resistance to these chemicals in mosquitoes. Therefore we need to identify alternative insecticide substances from natural products. Many scientists reported insecticidal activities of plants belong to different families in different parts of the world. There are several native reports about crude solvent extracts of different parts of plants, essential oils or their chromatographic fractions. They showed various levels of bioactivity against different developmental stages of malaria vectors (44). Some plants have phyto-chemicals constituents for the control of mosquitoes. One of the earliest reports of the use of plant extracts against mosquito larvae is extraction of plants’ alkaloids like nicotine, anabasine, methyl anabasine and lupinine from the Russian weed in 1933 (45). Some plant families such as *Asteraceae*, *Cladophoraceae*, *Labiatae*, *Meliaceae*, *Oocystaceae* and *Rutaceae* have the maximum potential for development of novel mosquito control agents (46). The genus *Lawsonia* has one species, *Lawsonia inermis* (47–48)*.* Henna`s leaves, flowers, seeds, stem barks and roots had been used in Iran to treat some diseases such as rheumatoid arthritis, headache, ulcers, diarrhea, leprosy, fever, leucorrhoea, diabetes, cardiac disease. It had hepatoprotective effect and been used as colouring agent too (49).

## Conclusion

Due to larvicidal effect of some Iranian native plants against malaria vector, production of specific formulation is required for evaluation under filed condition.
